# Contribution of viral and bacterial infections to senescence and immunosenescence

**DOI:** 10.3389/fcimb.2023.1229098

**Published:** 2023-09-11

**Authors:** Antonia Reyes, Gerardo Ortiz, Luisa F. Duarte, Christian Fernández, Rosario Hernández-Armengol, Pablo A. Palacios, Yolanda Prado, Catalina A. Andrade, Linmar Rodriguez-Guilarte, Alexis M. Kalergis, Felipe Simon, Leandro J. Carreño, Claudia A. Riedel, Mónica Cáceres, Pablo A. González

**Affiliations:** ^1^ Millennium Institute on Immunology and Immunotherapy, Santiago, Chile; ^2^ Facultad de Ciencias Biológicas, Pontificia Universidad Católica de Chile, Santiago, Chile; ^3^ Facultad de Ciencias de la Vida, Universidad Andres Bello, Santiago, Chile; ^4^ Program of Cellular and Molecular Biology, Institute of Biomedical Sciences, Faculty of Medicine, Universidad de Chile, Millennium Nucleus of Ion Channel-Associated Diseases (MiNICAD), Santiago, Chile; ^5^ Programa de Inmunología, Instituto de Ciencias Biomédicas, Facultad de Medicina, Universidad de Chile, Santiago, Chile; ^6^ Departamento de Endocrinología, Facultad de Medicina, Pontificia Universidad Católica de Chile, Santiago, Chile

**Keywords:** senescence, immunosenescence, chronic infections, persistent infections, virus, bacteria, SASP

## Abstract

Cellular senescence is a key biological process characterized by irreversible cell cycle arrest. The accumulation of senescent cells creates a pro-inflammatory environment that can negatively affect tissue functions and may promote the development of aging-related diseases. Typical biomarkers related to senescence include senescence-associated β-galactosidase activity, histone H2A.X phosphorylation at serine139 (γH2A.X), and senescence-associated heterochromatin foci (SAHF) with heterochromatin protein 1γ (HP-1γ protein) Moreover, immune cells undergoing senescence, which is known as immunosenescence, can affect innate and adaptative immune functions and may elicit detrimental effects over the host’s susceptibility to infectious diseases. Although associations between senescence and pathogens have been reported, clear links between both, and the related molecular mechanisms involved remain to be determined. Furthermore, it remains to be determined whether infections effectively induce senescence, the impact of senescence and immunosenescence over infections, or if both events coincidently share common molecular markers, such as γH2A.X and p53. Here, we review and discuss the most recent reports that describe cellular hallmarks and biomarkers related to senescence in immune and non-immune cells in the context of infections, seeking to better understand their relationships. Related literature was searched in Pubmed and Google Scholar databases with search terms related to the sections and subsections of this review.

## 1 Introduction

Although the notion of senescence dates back to pre-historic ages, the concept of cellular senescence was described more recently in 1961 by Leonard Hayflick and Paul Moorhead while studying human fibroblasts (Hayflick and Moorhead, 1961). The principle was described as a stable state resulting from the exit of the cell-division cycle process, and characterized by a finite proliferative capacity of human fibroblasts in cultures (Hayflick and Moorhead, 1961; Hayflick, 1965). Since then, the definition of this concept has evolved to be commonly known as a cellular stress response that induces stable cell-cycle arrest in previously replication-competent cells, while maintaining a normal metabolic activity and viability (Muñoz-Espín and Serrano, 2014). However, senescence is accompanied by a wide range of intrinsic and extrinsic cellular alterations, such as chromatin remodeling, increased autophagic activity, mitochondrial dysfunction, oxidative and genotoxic stress and the presence of a complex pro-inflammatory secretome which may relate to detrimental tissue performance, although senescence also displays beneficial aspects which are discussed below (Herranz and Gil, 2018; McHugh and Gil, 2018). Additional distinctive features of senescence include the activation of damage-sensing signaling pathways, such as p38^MAPK^ and NF-κB, the expression of antiproliferative molecules, such as p16, p21^cip1^, increased senescence-associated β-galactosidase (SA-β-Gal) activity, and often DNA damage, among others (Muñoz-Espín and Serrano, 2014; He and Sharpless, 2017).

Even though the term senescence was initially described for fibroblasts, several other cell types displaying senescence-related features were later reported, such as kidney cells, peripheral blood T cells and skin cells (Dimri et al., 1995; Melk et al., 2004; Liu et al., 2009). The induction of senescence and how senescence relates to different cellular processes has been extensively reviewed by Mohamad Kamal et al. (Mohamad Kamal et al., 2020), namely: (i) stress-induced premature senescence, including cellular senescence triggered by oxidative and genotoxic stresses, (ii) replicative senescence induced by telomere-shortening, and (iii) developmental senescence that occurs in a programmed manner during normal embryogenesis.

Importantly, it has been described that senescence has contrasting roles and effects in an individual’s physiology, as it may cause beneficial or detrimental effects depending on the circumstances (Muñoz-Espín and Serrano, 2014). For instance, transient induction of senescence in endothelial and fibroblast cells has been reported to contribute to wound healing (Jun and Lau, 2010; Demaria et al., 2014). Furthermore, senescence acts as a potent physiological anti-tumor mechanism, as it inhibits the development of malignancies by limiting the replication of preneoplastic cells (Collado and Serrano, 2010; Sharpless and Sherr, 2015). Additionally, senescence plays a pivotal role as a positive regulator in tissue remodeling and repair during the development stage of organisms and adulthood (Demaria et al., 2014; Muñoz-Espín and Serrano, 2014). Moreover, senescence has been linked to an attenuation of liver fibrosis, reduced skin scarring and oral fibrosis, mitigation of renal fibrosis, and protection against atherosclerosis, among others (Muñoz-Espín and Serrano, 2014).

On the other hand, the accumulation of senescent cells may lead to age-related diseases like cataracts, osteoarthritis, diabetes, and dementia among others (Mylonas and O’Loghlen, 2022).For example, idiopathic pulmonary fibrosis has been reported to be aggravated by senescence (Muñoz-Espín and Serrano, 2014; Mylonas and O’Loghlen, 2022). Additionally, senescent adipocytes are associated with obesity, and senescence contributes to type-2 diabetes (Fülöp et al., 2016). Furthermore, senescence worsens the outcome of sarcopenia and exacerbates cataracts (Muñoz-Espín and Serrano, 2014). Noteworthy, the immune system is also affected by aging, and its dysregulation and deterioration can be referred to as immunosenescence (Fülöp et al., 2016). Immunosenescence can predispose senior individuals to an impaired response to infections upon encounter with pathogens, the development of autoimmune disorders, as well as chronic non-immune disorders, including cardiovascular and neurodegenerative diseases, cancers, and type- 2 diabetes (Simon et al., 2015; Fülöp et al., 2016). Importantly, the study of senescence and immunosenescence in the context of infections is a growing field with a significant number of new reports emerging on a monthly basis, highlighting the increasing interest in this field, as it adds on to the intricate relationship between host and pathogens, altogether unveiling potentially new targets to counteract infections and related diseases. Here, we revise and discuss current and recent evidence regarding the interplay between immunosenescence and infections and provide a view on important questions remaining to be addressed in the field.

## 2 Methods

Pubmed from the U.S. Department of Health and Human Services (HHS) and Google Scholar databases were used to search for published studies using the following terms: a. For the section related to cellular and molecular biomarkers related to senescence: biomarkers and cellular senescence, biomarkers and senescence, molecular biomarkers and hallmark of cellular senescence. b. For the sections related to infections and immunosenescence: viruses and senescence, viruses and immunosenescence, RNA viruses and senescence, RNA viruses and immunosenescence, DNA viruses and senescence, DNA viruses and immunosenescence, bacteria and senescence, bacteria and immunosenescence. c. For the section related to immunosenescence: immunosenescence, T cells and senescence, T cell immunosenescence, B cells and senescence, B cell immunosenescence, innate immune system and senescence, adaptative immune system and senescence. Articles were included in the review when they matched the search parameters and provided new information for the article.

## 3 Cellular and molecular biomarkers related to senescence

Relating senescence to infections will require using markers that adequately mark for senescence. Yet, senescence does not have a unique molecular marker, but rather multiple determinants that relate to this cellular state have been identified. Hence, numerous markers may be used, and frequently more than one will be applied to corroborate this phenotype. However, some are more widely utilized than others. Among the different types of senescence, replicative senescence was one of the first types to be described (Hayflick and Moorhead, 1961). This type of senescence, evidenced in cell culture, occurs in response to continuous and successive cell divisions and is characterized by a decrease in the length of telomeres and, consequently an increase in DNA damage (d’Adda di Fagagna et al., 2003). Yet, whether pathogens eliciting senescence in infected cells inhibit cell proliferation or not has, in our opinion, has not been fully addressed and may be highly dependent on the pathogen, with some seemingly not interfering with this process and others considered as risk factors for this condition, such as retroviruses (Dolan et al., 2006; Stone et al., 2010). Some pathogens will hijack the cell and its functions redirecting all cellular resources to pathogen replication, thus hampering cellular division (Noris et al., 2002; Zannetti et al., 2006; Bartkova et al., 2006; Nikitin et al., 2014). The varied nature of senescence markers may also relate to the fact that senescence can be induced by several different types of stimuli, such as oxidative stresses (Hernandez-Segura et al., 2017), mitochondrial dysfunctions (Wiley et al., 2016), activation of oncogenes (Muñoz-Espín and Serrano, 2014; Sharpless and Sherr, 2015), chemotherapeutic agents (Petrova et al., 2016), and cytokines (Acosta et al., 2013), among others (Campisi and d’Adda di Fagagna, 2007; Kuilman et al., 2010; Salama et al., 2014; van Deursen, 2014), with each eliciting particular senescence-related features that not necessarily overlap under all circumstances. This is particularly relevant, as in the context of infections, it is possible that viruses, bacteria and parasites induce senescence and classical biomarkers of senescence (Dodig et al., 2019), which can be further broken down depending on the species of each of these microorganisms. Yet, this remains to be determined.

**Figure 1:**
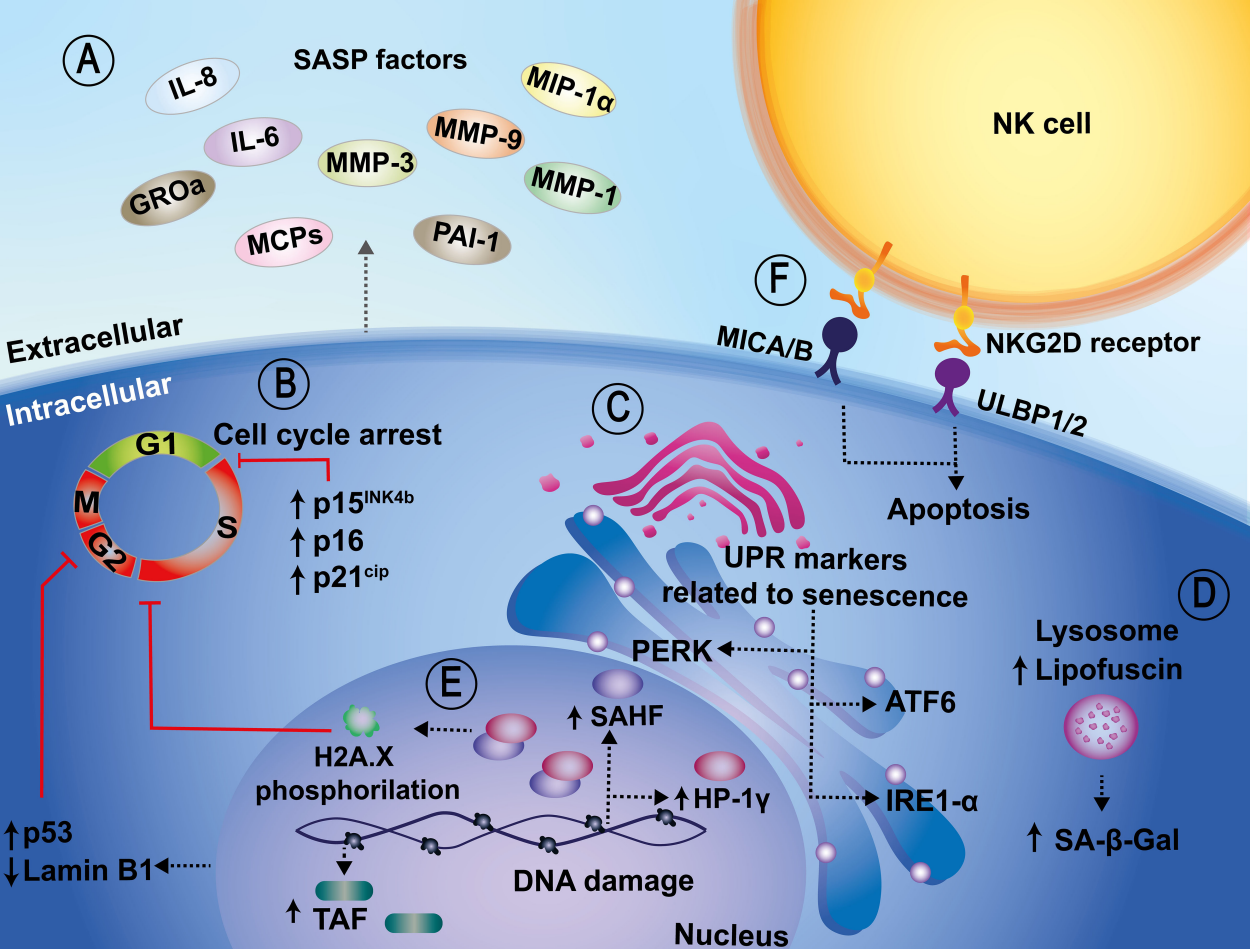
Cellular and molecular biomarkers related to senescence. **(A)** Senescent cells show characteristic transcriptional alterations, such as the acquisition of a pro-inflammatory secretome, known as the senescence-associated secretory phenotype (SASP), which relates to IL-6, IL-8, GROa, MCPs, MMP-1, MMP-3, MMP-9, MIP-1α, and PAI-1 release, among others. **(B)** An increase in senescence-associated beta-galactosidase (SA-β-Gal) activity, which is also a senescence marker, has been associated with upregulated expression of cyclin-dependent kinase inhibitors, such as p15^INK4b^, p16, p21^CIP1^, p53. These in turn induce cell cycle arrest. **(C)** Other additional biomarkers include sensors of the unfolded protein response (UPR), such as IRE1-α and ATF6. **(D)** Another marker related to senescence is the accumulation of lipofuscin at the lysosomal level. Lipofuscin and SA-β-Gal are colocalized in senescent cells. **(E)** In the nucleus, increased redistribution of senescence-associated heterochromatic foci (SAHF), telomere-associated DNA damage foci (TAF), and downregulation of Lamin B1 are also considered markers of senescence. **(F)** Lastly, the NKG2D/NKG2DL interaction promotes the activation of the cytotoxic activity of NK cells and the induction of apoptosis in senescent cells.

Although the mechanisms eliciting cellular senescence vary depending on the stimuli, nevertheless there are many signaling pathways that coincide or converge, such as the activation of p53 (encoded in humans by the *TP53* gene and in mice by the *Trp53* gene), which leads to the downstream induction of cell cycle arrest through the activation of cyclin-dependent kinase (CDK) inhibitors p16 (known as *INK4a*, encoded by *CDKN2A* gene), p15 (*INK4b*, encoded by *CDKN2B* gene), p21 (also known as WAF1, encoded by *CDKN1A* gene) and p27 (encoded by *CDKN1B* gene) (Muñoz-Espín and Serrano, 2014) (**Figure 1**).

Senescent cells display unique morphological, molecular, and biochemical features, as well as functions that differentiate them from other non-dividing cell populations, such as quiescent and terminally-differentiated cells (Roger et al., 2021). However, cellular infection is frequently followed by morphological changes, commonly known as cytopathic effects, and thus this parameter may be hard to apply in the context of host-pathogen interactions. Interestingly, within senescent cells there are also characteristic transcriptional changes, the acquisition of a pro-inflammatory secretome (SASP), macromolecular damage, and deregulated metabolism (Hernandez-Segura et al., 2018; Gorgoulis et al., 2019; Roger et al., 2021). However, once again, because infections elicit profound changes in cellular transcription due to the activation of signaling pathways that might detect pathogen associated molecular patterns (PAMPs), it may be hard to differentiate host pathogen recognition receptor (PRR)-induced inflammatory responses from senescence-related cytokine responses.

Because senescent cells can be detrimental to the individual, immune cells can detect and selectively eliminate them by recognizing specific surface molecules expressed on these cells (Kale et al., 2020). Some reports indicate that the major histocompatibility complex (MHC) class I chain-related protein A and B (MICA/B), and UL16 binding proteins 1 and 2 (ULBP1/2) are upregulated in senescent cells, allowing the recognition of these cells by NKG2D receptors expressed on the surface of Natural Killer cells (NK cells) (**Figure 1**) (Sagiv et al., 2016). Importantly, the NKG2D/NKG2DL interaction promotes the activation of the cytotoxic activity of NK cells and the induction of apoptosis in senescent cells (Sagiv et al., 2016).

However, the mechanism by which senescent cells regulate their immune surveillance has yet to be completely understood. Below, are described biomarkers related to senescence that can lead to their recognition by immune cells and subsequent elimination. However, several senescence markers are intracellular and not visible to immune cells.

### 3.1 Biomarkers reported in senescent cells

A biomarker is a biological indicator that may be used alone or in combination with others to identify a particular cell types or biological conditions. These may consist in different types of biomolecules, including proteins, nucleic acids, carbohydrates, and lipids (Matjusaitis et al., 2016). Below, we evoke different biomarkers associated with senescence that help identify these cells. It is important to keep in mind that although some of these hallmark features relate to senescence, they also connect to other cellular processes and cellular states and thus, to date, there is still some difficulty in defining a particular set of markers that allow full recognizing and distinguishing senescent cells from other cell types (Sharpless and Sherr, 2015; Wang and Dreesen, 2018). Indeed, no single biomarker currently provides a reliable and precise representation of a senescent cell phenotype (Matjusaitis et al., 2016).

At present, the most reliable indicators of senescent cells in cell cultures, or tissue samples are those related to the expanded and flattened shape of cells, together with enhanced activity SA-β-Gal, which is considered a gold-standard marker for the identification of these types of cells (Debacq-Chainiaux et al., 2009). The drawback of this method is that it requires assessing the enzymatic activity, which is preferentially preserved in fresh tissues and/or samples, and may be lost in samples that have been subjected to fixation or cold preservation (Severino et al., 2000; Wang and Dreesen, 2018). Another marker related to senescence at the lysosomal level is the accumulation of the “age pigment” lipofuscin, which is a non-degradable aggregate consisting of oxidized proteins, lipids, and metals (Jung et al., 2007; Georgakopoulou et al., 2013). There is evidence that lipofuscin and SA-β-Gal are colocalized in senescent cells both, *in vitro* and *in vivo*, which strongly supports the idea that lipofuscin may be a valuable biomarker of cellular senescence (Georgakopoulou et al., 2013) (**Figure 1**). However, viral infections frequently involve altered dynamics of cellular organelles and compartments, and thus alterations in the localization of host proteins within the cell might occur (Glingston et al., 2019). Other biomarkers include sensors of the unfolded protein response (UPR), such as IRE1α, ATF6 and PERK, which are activated in senescent cells, indicating that senescence may be induced by an imbalance and alterations in cellular proteostasis (Abbadie and Pluquet, 2020). Indeed, the UPR may be a critical process involved in the senescence phenotype; however, there are significant nuances and particularities in how the many complex UPR pathways may be involved (Abbadie and Pluquet, 2020) (**Figure 1**). Nevertheless, given the relevance of this pathway, cell infection with microorganisms may lead to the activation or inhibition of these pathways as part of the infectious pathogen cycle, as reviewed by Vibhu Prasad et al. (Prasad and Greber, 2021).

During oncogene-induced senescence (OIS) *in vitro*, there are changes at the chromatin level as heterochromatin is redistributed into senescence-associated heterochromatin foci (SAHF), which co-localize with the heterochromatin protein 1γ (HP-1γ protein) which is related to transcriptional repression of genes associated with cell proliferation (Chen et al., 2007). The gold-standard marker for senescence is the phosphorylation of histone H2A.X at serine139 (γH2A.X), (Saldías et al., 2017; Fernandez et al., 2021). Concurrent with the redistribution of heterochromatin, telomere-associated DNA damage foci (TAF) have also been used as a senescence marker (Rossiello et al., 2022). Interestingly, these senescence biomarkers have been less explored in the context of infections and thus, it will be relevant to determine if they arise during the interaction of microorganisms with target cells.

In addition, the downregulation of Lamin B1 in cells undergoing different sublethal stimuli *in vitro* has also been reported and used as a biomarker of cellular senescence (Wang and Dreesen, 2018) (**Figure 1**). This latter biomarker can result particularly challenging considering that some pathogens, namely some types of viruses, alter or disrupt nuclear integrity upon infection as a mechanism to extend their genomes from this compartment onto the cytoplasm on their way out of the cell. This is particularly relevant for pathogens such as retroviruses (De Noronha et al., 2001; Takeshima et al., 2022; Wen et al., 2022).

Finally, senescent cells can develop a which has been used to detect and assess senescence in cultured cells or tissue samples. It comprises inflammatory mediators, growth factors, proteases, soluble surface molecules, and extracellular matrix components, among others (Coppé et al., 2010). However, the drawback of the secretory profile of senescent cells is that it varies depending on the cell type and the stage of senescence and in the context of cell infection will likely be overlapped with profiles of soluble molecules being secreted as a result of infection (Coppé et al., 2008; Campisi et al., 2011; Acosta et al., 2013) (**Figure 1**). Thus, defining which components of the secreted molecules relate to SASP, and which are inherent to a cellular response to pathogens will be relevant to identify senescence in the context of infections and assess the contribution of this latter process in the host response to harmful microorganisms.

As revised above, senescence relates to numerous cellular markers that overlap with host responses to infection and thus, defining a state of senescence in such cells can be particularly challenging. Undoubtedly, while adaptive immune cells, and to a lesser extent innate immune cells, may experience a process called immunobiography which corresponds to a reduction or a cessation of their defensive functions in cases of prolonged, chronic, or recurrent infections, they may also undergo senescence as a response to these persistent infections. This senescence can serve as a mechanism to signal and enhance the recruitment of immune cells (Franceschi,et al., 2017) or that pathogens are favored by cellular senescence phenotypes in target cells, assuring cell viability, yet at the potential cost of the infected cells being recognized by host immune components. These scenarios remain to be addressed in future studies.

## 4 Interplay between systemic infections and immunosenescence

Due to the relevance of senescence in the biology of cells and its role in tissue fitness, studying its participation and interrelationship with infections has become a growing field of research. Interestingly, cell infection with microorganisms has been reported to elicit the expression of numerous senescence biomarkers as those discussed above. An important question is whether pathogens induce senescence in infected cells, or alternatively, induce the expression of molecular determinants that coincide with those associated with this cellular process. Either way, studying the presence of these molecular markers in infected cells and their relationship, or coincidence with senescence markers will undoubtedly expand our knowledge on senescence *per se* and infections. Below, we discuss senescence an immunosenescence in the context of infections, with the latter defined as senescence processes in immune system cells as individuals undergo aging. As further discussed below, immunosenescence relates to a series of complex changes leading to altered innate and adaptive immune system immune functions, which overall may result in a state of immunodeficiency (Akbar and Fletcher, 2005; Nikolich-Žugich, 2018).

### 4.1 Immunosenescence and RNA viruses

Since late 2019, a considerable amount of attention from the biomedical field has focused on SARS-CoV-2 and the emergence of its related respiratory infectious disease termed coronavirus disease 2019 (COVID-19), which after identified was rapidly considered a somewhat systemic-related disease in an important fraction of individuals together with a respiratory disease (Maldonado et al., 2022). Interestingly, it has been reported that T cells from patients with COVID-19 display a senescent phenotype and exhaustion, which is associated with exacerbated cytokine production (De Biasi et al., 2020). Also, the function of CD4^+^ and CD8^+^ T cells was severely altered in patients with this disease, displaying a senescent phenotype involved in severe disease manifestations (Zuin et al., 2022). Furthermore, a study reported senescent and hyperinflammatory phenotypes in nonimmune cells obtained from patients who died from COVID-19 (Evangelou et al., 2022). Moreover, the same study showed that senescence promoted DNA damage in SARS-CoV-2-infected Vero-E6 cells and increased the expression of the apolipoprotein B mRNA-editing enzyme, which plays a role in virus mutagenesis, likely enhancing viral pathogenicity and persistence (Evangelou et al., 2022).

Lung epithelial and endothelial cells obtained from tissue samples from sick COVID-19 patients evidenced that the spread of a senescent phenotype was related to increased disease susceptibility and severity (D’Agnillo et al., 2021), consistent with an increased risk in older adults or people with underlying diseases (D’Agnillo et al., 2021). Moreover, in an aged mouse model of SARS-CoV-2-induced pneumonia, senescence was also promoted, especially in macrophages, which displayed abnormal activation of the STING/NLRP3 inflammasome pathway, seemingly by mitochondrial DNA leakage and animals predisposed to severe inflammation (Lv et al., 2022). Interestingly, mitochondrial integrity, macrophage-associated burden, and the course of viral infection were improved by using senolytic drugs, that selectively cleared senescent cells, suggesting that exacerbated inflammatory response and increased susceptibility to infection is regulated by senescence in mice deficient in telomerase RNA Terc^-/-^ mice (Lv et al., 2022).

**Figure 2:**
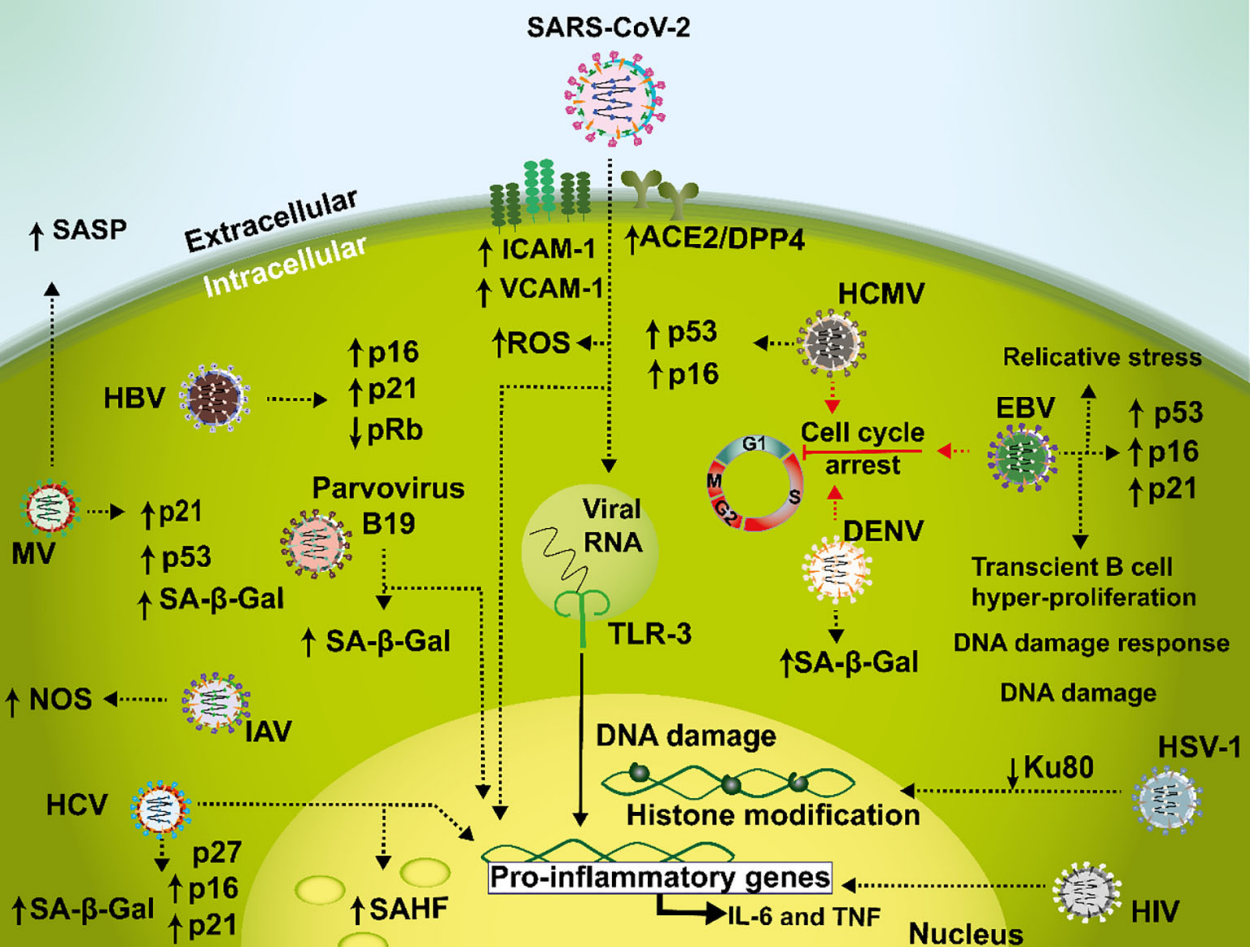
Mechanisms mediating senescence interplay with viral infections. During SARS-CoV-2 infections, a senescent phenotype is characterized by a pro-inflammatory environment involved in a dysfunctional loop with increased ACE2/DPP4 receptor expression leading to an induction in ICAM-1, VCAM-1 and ROS. In addition, TLR-3 amplifies this feedback during prolonged infection, senescence, and hyperinflammation. Hepatitis C virus (HCV) causes an increase in p16, p21, and p27, which cause cell cycle arrest in liver tissue samples and in hepatocellular carcinoma. Cellular senescence is also associated with HIV disease where this virus may induce IL-6 and TNF during infection. Measles virus (MV) is capable of generating cellular senescence as evidenced by reduced cell proliferation, SA-β-Gal activity, increased expression of p53 and p21 and induction of SASP in IMR90 cells. In HUVEC cells, Dengue virus elicits SA-β-Gal expression and cell cycle arrest. Furthermore, influenza A virus (IAV) has been described to induce cellular senescence related to an increased expression of NOS in Neuro2a cells. Human cytomegalovirus (HCMV) induces senescence through activation of p53 and p16 in human fibroblasts. Additionally, in B cells Epstein-Barr virus (EBV) triggers a G1 cellular arrest phase and an increase of p16, p21 and p53. Herpes simplex virus type 1 (HSV-1) infection leads to DNA damage trough a reduction of Ku80. Hepatitis B virus (HBV) infection is related to an increased expression of p16 and p21 and a decreased expression of pRb in malignant liver cell lines. Lastly, parvovirus B19 induces cellular senescence as seen by an increase in SA-β-Gal activity in human dermal fibroblasts.

It has been reported that severe COVID-19 is also characterized by hemostatic alterations that promote thrombotic complications (Meyer et al., 2021). SARS-CoV-2-induced senescence in endothelial cells has been described to be accompanied by an increase in pro-inflammatory cytokines, reactive oxygen species (ROS), as well as endothelial adhesion molecule expression Intercellular Adhesion Molecule 1 (ICAM-1) and Vascular Cell adhesión Molecule 1 (VCAM-1), and enhanced leukocytes attachment (Meyer et al., 2021). Interestingly, those features were also prevented with senolytic agents, pointing out a critical role for senescence, and highlighting its therapeutic potential to attenuate COVID-19-elicited adverse symptoms (**Figure 2**).

Current evidence indicates that the relationship between senescence and the dysfunctional inflammatory response induced by SARS-CoV-2 is far from being unidirectional. A recent report (Kandhaya-Pillai et al., 2022) suggests positive feedback between the inflammatory cytokines TNF-α and IFN-γ, and the induction of a senescent phenotype, decreasing cellular proliferation capacity in primary endothelial cells and increasing the expression of the SARS-CoV-2 receptors ACE2/DPP4 through the JAK/STAT1 pathway (Kandhaya-Pillai et al., 2022). All these findings support a feedback loop between prolonged infection, hyperinflammatory states, and a senescent status. In addition, Toll-like receptor (TLR) 3 has recently emerged as an uncovered participant involved in initiating and amplifying SARS-CoV-2-induced senescence, increasing the inflammatory burden and worsening the host response in COVID-19 (Tripathi et al., 2021) (**Figure 2**).

Several other viral infections have also been described to be associated with senescence, for example, hepatitis C virus (HCV) which infects more than 170 million people worldwide and is linked to liver inflammation and diseases, such as diabetes mellitus, cirrhosis, and fatty liver disease (Serfaty and Capeau, 2009; Malnick et al., 2014). Studies with this virus suggest that the progression of liver fibrosis is closely associated with senescence in parenchymal and non-parenchymal liver cells during hepatitis and liver-associated diseases, such as hepatic steatosis and non-alcoholic liver disease elicited by this virus (Marshall et al., 2005; Aravinthan et al., 2013; Wandrer et al., 2018). Interestingly, it has been reported that the expression of CDK inhibitors p21, p27, and p16 is increased in liver tissues obtained from patients with chronic HCV infection, as compared to livers from healthy individuals (Bassiouny et al., 2010; Wandrer et al., 2018). HCV infection is also associated with senescence-related epigenetic alterations recruited at SAHF, such as the phosphorylation of HP-1γ in liver tissue samples and the histone γH2A.X in peripheral blood mononuclear cells (PBMCs), which are known to be markers of senescence (Hoare et al., 2013; Wandrer et al., 2018) (**Figure 2**). Furthermore, tissue samples obtained from patients at different stages of infection with HCV-related fibrosis showed a higher proportion of p-HP-1γ and γ-H2AX positive cells during chronic HCV than healthy controls (Wandrer et al., 2018) (**Figure 2**). These senescence markers were more expressed along fibrotic scars, whereas only a few senescent cells were found in the liver parenchyma (Krizhanovsky et al., 2008; Wandrer et al., 2018). Moreover, *in vitro* analyses with HepG2 cells have shown a direct association between hepatocyte-related senescence and liver fibrosis caused by HCV. This association is related to the senescence markers SA-βgal and IL-1β, which are also highly expressed in non-alcoholic fatty liver disease and alcoholic liver disease (Wijayasiri et al., 2022).

HCV chronic hepatitis has been reported to be capable of accelerating telomere shortening in hepatocytes infected by this virus. This promotes the accumulation of senescent cells, which shows a strong correlation with increased hepatic fibrosis in hepatocellular carcinoma (HCC) and the overexpression of p16 and p21 (Paradis et al., 2001; El-Serag and Rudolph, 2007; Desai et al., 2019) (**Figure 2**). Additionally, it has been proposed that the number of senescent cells in the liver reflects the progression of fibrosis in patients with chronic hepatitis C, evidenced by the presence of senescence markers in non-parenchymal fibrotic tissue, and to a higher proportion of intrahepatic senescent T cells (Wandrer et al., 2018; Giannakoulis et al., 2021). Noteworthy, these senescent T cells are not functional. Yet, they are abundant during *in vivo* viral infection (Voehringer et al., 2001). Thus, overall, HCV infection likely elicits an increase in senescent T cells that may predispose the individual to the development of HCC, as these T cells would not able to eliminate premalignant senescent hepatocytes (Kang et al., 2011).

Another scenario that connects virus and senescence is osteoporosis and osteopenia, in which the human immunodeficiency virus (HIV) increases the likelihood of these senescence-related diseases (Ofotokun et al., 2012; Olali et al., 2022). Cellular senescence is considered to be an important factor in this disease, and there is compelling evidence that HIV infection is a risk factor for this pathological condition (Dolan et al., 2006; Stone et al., 2010). There is evidence that HIV may increase bone turnover by itself and that it may be associated to an increase in cytokine expression, such as IL-6 and TNF during infection (Dolan et al., 2006) (**Figure 2**). However, the mechanisms underlying this phenomenon are yet to be elucidated (Dolan et al., 2006). Interestingly, it has been shown in individuals living with HIV and co-infected with HCV that the spontaneous clearance of the latter corelates with a slightly lower senescence profile (Lara-Aguilar et al., 2023).

Another example is measles virus (MV), which when infecting human lung fibroblast cells (IMR90 cells) is capable of generating cellular senescence as evidenced by reduced cell proliferation, SA-β-Gal activity, increased expression of p53 and p21, or the induction of SASP with interleukin-8 (IL-8) or C-C motif chemokine ligand 5 chemokine ligand 5 (CCL5) expression (Seoane et al., 2020) (**Figure 2**). In addition, MV infection has also been related to triggering senescence in the A549 adenocarcinoma cell line, in a p53-dependent manner (Seoane et al., 2020).

On the other hand, dengue virus (DENV) when infecting human umbilical vein endothelial cells (HUVEC) has been reported to elicit SA-β-Gal expression, cell cycle arrest and morphological changes are typical of senescent cells (**Figure 2**). It has been proposed that this process contributes to the pathogenesis of the virus. However, whether DENV-induced senescence occurs through a DNA damage-mediated pathway is unknown at this time (AbuBakar et al., 2014).

Finally, in some strains of influenza A virus (IAV), the non-structural protein NS1 which is related to the evasion of the host antiviral response and interference with the translation and maturation of interferon mRNAs, has been shown to be capable of generating cellular senescence (Yan et al., 2017). In a fast-growing mouse neuroblastoma cell line (Neuro2a cells) and in mouse cortical cells neurons, the NS1 protein of IAV strain H7N9 was described to induce cellular senescence related to an increase in the expression of the nitrous oxide synthase enzyme, which in turn lead to a higher release of nitric oxide (Yan et al., 2017) (**Figure 2**). Recently, it has been shown, both in an *in vitro* model of infected human monocyte-derived macrophages on primary lung fibroblasts, and *in vivo* in 5-week-old female BALB/cJRj-wild-type mice that infections with IAV lead to premature cellular senescence through paracrine induction of TNF-α, and that subsequent infections lead to an increased viral replication (Schulz et al., 2023).

Although vaccines are directly related to increased quality of life and extended living of individuals, it seems that studies evaluating senescence and immunosenescence in the context of infections, and their impact on long-term health, are inexistant likely due to our constant exposure to pathogens and thus, the related difficulties to register all these events during extended periods. Nevertheless, it may ultimately be possible to envisage, to some extent, the assessment of the impact of the exposure to some particular pathogens for which there are better registries, such as COVID-19 during the SARS-CoV-2 pandemic, with overall cell aging. Given the cellular nature of senescence and immunosenescence, a relationship could eventually be determined in blood cells or tissue biopsies. In this sense, it will be interesting to assess whether extended health problems due to infection, such long COVID, or long-term effects produced by some respiratory viruses such as the respiratory syncytial virus or influenza virus, are related to senescence or immunosenescence.

### 4.2 Senescence and DNA viruses

A persistent latent infection caused by the herpesvirus Kaposi’s sarcoma virus (KSV), promotes the proliferation of lymphatic endothelial cells and senescence, depending of the ratio between bFLIP and vCyclin (DiMaio et al., 2020). Human cytomegalovirus (HCMV) another herpesvirus is also an example of a virus in which the generation of senescence occurs with a mechanisms being proposed through the activation of p53, as well as p16 in primary human embryo lung fibroblasts and adult diploid fibroblasts (Noris et al., 2002; Zannetti et al., 2006, 16) (**Figure 2**). When fibroblasts are infected with HCMV, this virus induces premature senescence and cell cycle arrest, likely mediated by the expression of the viral immediate-early 2 (IE2) protein (Ball et al., 2022). Additionally, there is evidence that indicates that the expression of other individual viral proteins may also promote cellular senescence, similar to the HCMV IE2 protein (Noris et al., 2002; Ball et al., 2022). Moreover, aging and viral infections seemingly influence HCMV-induced immune alterations that impair CD8^+^ T cell immunity later in life in a dose-dependent manner, which is related to HCMV-associated immune senescence (Redeker et al., 2017). Noteworthy, it has been reported in human diploid fibroblast WI-38 cells that treatment with pterostilbene, a stilbenoid chemically related to resveratrol, reduces viral infection and has an inhibitory effect over molecular determinants related to cellular senescence and reactive oxygen species produced by this virus (Wang et al., 2022).

It has been reported that Epstein-Barr virus (EBV), also a herpesvirus, induces replicative stress, DNA damage and activation of DNA damage response in cells, as well as cellular senescence by inducing transient B cell hyper-proliferation through upregulation of the viral latency proteins, such as EBNA2 and EBNA-LP (Nikitin et al., 2010; Hafez and Luftig, 2017). Moreover EBV infection causes metabolic reprogramming including decreased oxidative phosphorylation and purine nucleotide pools contributing to increased replication stress and persistent DNA damage as seen in B cells obtained from PBMCs (Hafez et al., 2017). EBV infection has also been seen to induce the expression of senescence-associated markers, such as KLRG-1 as seen in antigen-specific T cells isolated from peripheral blood of EBV-infected patients (Lanfermeijer et al., 2021). Additionally, it has been reported that EBV-infected B cells trigger a G1 cellular arrest phase and exhibit augmented markers of OIS, such as H3K9me3 senescence-associated heterochromatic foci, p16, p21 and p53 in (Nikitin et al., 2010; Nikitin et al., 2014; McFadden et al., 2016) (**Figure 2**). Recently, it has been reported that co-infection with EBV and HCMV in young and middle-aged adults can alter T cell composition towards an aging-related T cell phenotype (Hofstee et al., 2023).

Interestingly, a relationship between aging and age-related pathologies, including neurodegenerative diseases, such as Parkinson’s, multiple sclerosis (MS), and Alzheimer’s disease (AD), has been associated with herpes simplex virus (HSV) infections (Duarte et al., 2019; Reyes et al., 2022). Several studies have reported a high prevalence of HSV-1 DNA in the brains of patients with MS and senile plaques of individuals with AD, which contain amyloid beta (Aβ) peptide and phosphorylated tau protein depositions in neurons (BergstOum et al., 1989; Miklossy, 2011). Additionally, in cortical neurons, HSV-1 infection leads to the accumulation of DNA lesions, including single- and double-strand breaks (SSBs and DSBs, respectively) (**Figure 2**), which have recently been implicated in neuronal loss related to neurodegenerative diseases (De Chiara et al., 2016). HSV has also been associated with the induction of neuro-inflammation and senescence in the brainstem of female C57BL/6J mice, as observed by an increase in SA β-Gal activity, p16, p21 and p53 RNA levels (Sivasubramanian et al., 2022). The mechanisms behind this HSV-induced DNA damage remain to be elucidated. One report suggests that this is mediated by the downregulation of Ku80, a key component of non-homologous end joining (NHEJ). Ku80 is driven to proteasomal degradation affecting NHEJ, which produces DSB (De Chiara et al., 2016)

Furthermore, some studies associate HSV infections with neuronal aging, which could lead to AD. It has been shown, both *in vitro* and *in vivo*, that neurons increase the level of histone modifications that act as aging markers in response to HSV latent infection and reactivation, such as histone H4 acetylation at Lysine 16 (H4K16ac), the SIN3 histone-modifying complex, and the histone deacetylase 1 (HDAC1) (Napoletani et al., 2021). Additionally, the histone regulator A (HIRA) is upregulated during viral latency and has a different localization in cortical neurons obtained from HSV-1-infected mouse brains (Napoletani et al., 2021).

Hepatitis B virus (HBV) can elicit chronic infections closely related to liver cell senescence (Tachtatzis et al., 2015). HBV infection can induce senescence of hepatocytes and other liver cells by altering the hepatic tissue environment. In fact, pro-inflammatory and angiogenic factors eventually favor carcinogenesis in neighboring non-senescent cells (Brenner et al., 2013; Karakousis et al., 2020). Therefore, cellular senescence may play an important role in HBV-related hepatocarcinogenesis (Park et al., 2007; Brenner et al., 2013). Interestingly, the HBV protein x (HBx protein) exerts a pro-senescent role, evidenced by increased expression of p16 and p21^Waf1/Cip1^ in malignant liver cell lines, such as HepG2, Huh7 and SK-Hep1, and a decreased phosphorylation of the retinoblastoma protein (pRb), a tumor suppressor protein that is altered in many types of cancers (Weinberg, 1995; Sun et al., 1997; Morris and Dyson, 2001; Chen et al., 2007; Idrissi et al., 2016) (**Figure 2**). However, it has been reported that the effect of HBx on senescence is somewhat dependent on the cell type infected, as this viral protein promoted the proliferation of HepG2 malignant liver cells (Idrissi et al., 2016). Interestingly, a novel anti-HBV candidate drug consisting of a nucleos(t)ide analog namely E-CFCP, is able to rescue senescence-associated phenotypes in primary human hepatocytes infected with HBV (Takamatsu et al., 2023).

Merkel cell polyomavirus (MCPyV) has also been related to senescence. In normal human dermal fibroblasts (nHDF) transfected with replication-competent MCPyV, it was found that these cells increased β-galactosidase-related activity and arresting the cells in G_2_ (Siebels et al., 2020). Additionally, MCPyV infection induced cell cycle arrest in nHDF cells with ATM-dependent phosphorylation of Kap1 S824 (Siebels et al., 2020). Interestingly, in the same cell type it was shown that parvovirus B19 induces cellular senescence as seen by an increase in senescence markers, such as SA-β-Gal activity, DNA damage markers, morphological features and the expression of some SASP-related factors such as IL-8, IL-6 and IL-1β (Arvia et al., 2022) (**Figure 2**).

Compared to RNA viruses, significantly less vaccines are available to combat DNA viruses, which are mostly persistent in humans. Hence the effects described above for these viruses are likely cumulative throughout the life of an individual and constantly renewed in the context of sporadic reactivations. This has led to the consideration that some of these viruses likely relate to chronic diseases, namely neurodegenerative. Given the persistent nature and high prevalence of some of these DNA viruses, namely herpesviruses, it will likely result extremely hard to pinpoint their particular effects over individuals, as molecular reactivations can occur at single-cell level without visible symptoms (Grinde, 2013; Duarte et al., 2021). In such scenario, the contribution of only some viruses to senescence-related diseases may be identified given our currently available tools to detect infection-related processes (Duarte et al., 2023).

A summary of interplays between senescence and different viruses is outlined in **Table 1**.

**Table 1:** Interplay between viruses or bacteria with senescence.

Pathogen	Senescence interplay	References
*RNA viruses*		
**Severe acute respiratory syndrome coronavirus 2 (SARS-CoV-2)**	T cell with senescent phenotype	De Biasi et al., 2020
	Exacerbated cytokine production	De Biasi et al., 2020
	Senescence-induced DNA damage	Evangelou et al., 2022
	Increased expression of ApoB	Evangelou et al., 2022
	Abnormal activation of the STING/NLRP3 inflammasome pathway in macrophages	Lv et al., 2022
	Increase of pro-inflammatory cytokines, ROS, ICAM-1 and VCAM-1	Meyer et al., 2021
	Decreased cellular proliferation	Kandhaya-Pillai et al., 2022
	Increased expression of ACE2/DPP4	Kandhaya-Pillai et al., 2022
**Hepatitis C virus (HCV)**	Increased expression of p16, p21 and p27 in liver tissues of patient with chronic HSV infection and in hepatocellular carcinomas	Bassiouny et al., 2010; Paradis et al., 2001; Wandrer et al., 2018
	Phosphorylation of HP-1γ and the histone γH2A.X	Hoare et al., 2013; Wandrer et al., 2018
	Increased SA-β-Gal and IL-1β expression	Wijayasiri et al., 2022
**Human immunodeficiency virus (HIV)**	Related to osteoporosis and osteopenia	Ofotokun et al., 2012; Olali et al., 2022
	Increased UL-6 and TNF-α expression	Dolan et al., 2006
**Measles virus (MV)**	Reduced cell proliferation	Seoane et al., 2020
	Increased SA-β-Gal activity	Seoane et al., 2020
	Increased expression of p53 and p21	Seoane et al., 2020
	Induction of SASP, namely IL-8 and CCL5	Seoane et al., 2020
**Dengue virus (DENV)**	Increased SA-β-Gal activity	AbuBakar et al., 2014
	Induces cell cycle arrest	AbuBakar et al., 2014
	DENV-induced senescence through DNA damage	AbuBakar et al., 2014
**Influenza A virus (IAV)**	NS1 protein is capable of generating senescence through an increase in NOS	Yan et al., 2017
*DNA viruses*		
**Human cytomegalovirus (HCMV)**	Generation of senescence through the activation of p53 and p16^INK4a^	Ball et al., 2022
	Induces cell cycle arrest	Noris et al., 2002
**Epstein Barr virus (EBV)**	Induces replicative stress, DNA damage, DNA damage response and cellular senescence	Nikitin et al., 2010; Hafez and Luftig, 2017
	Decreased oxidative phosphorylation	Hafez et al., 2017
	Induces the senescence-associated marker KLRG-1	Lanfermeijer et al., 2021
	G1 cellular arrest and increases markers of OIS, such as H3K9me3 senescence-associated heterochromatic foci p16, p21 and p53	Nikitin et al., 2010; Nikitin et al., 2014; McFadden et al., 2016
**Herpes simplex virus (HSV)**	Related to several age-related pathologies, such as Parkinson’s, multiple sclerosis and Alzheimer’s disease	Duarte et al., 2019
	Induces the accumulation of DNA lesions	De Chiara et al., 2016
	Increases levels of histone modifications, such as histone H4 acetylation at Lysine 16 (H4K16ac), the SIN3 histone-modifying complex, and histone deacetylase 1 (HDAC1)	Napoletani et al., 2021
**Hepatitis B virus (HBV)**	HBVX exerts a pro-senescent role, as it induces the expression of p16 and p21^Waf1/Cip1^, and decreases the phosphorylation of pRb	Weinberg, 1995; Sun et al., 1997; Morris and Dyson, 2001; Chen et al., 2007; Idrissi et al., 2016
**Merkel cell polyomavirus (MCPyV)**	Increased SA-β-Gal-related activity and cell arresting at G2	Siebels et al., 2020
	Induces cell cycle arrest	Siebels et al., 2020
**Parvovirus B19**	Induces an increase in senescence markers, such as SA-β-Gal activity, DNA damage markers, morphological features and the expression of some SASP-related factors such as IL-8, IL-6 and IL-1β	Arvia et al., 2022
*Bacteria*		
***Streptococcus pneumoniae***	Increased senescence markers, such as p16, IL-1α/β, TNF-α, IL-6 and CXCL1	Shivshankar et al., 2011
***Fusobacterium nucleatum***	Caveolin-1 enhances infection	Ahn et al., 2017
***Escherichia coli***	Increased senescence markers such as PML bodies, SA-β-Gal activity and senescence-associated heterochromatic foci	Secher et al., 2013
***Mycobacterium tuberculosis***	Infected T cells express higher levels of KLRG1	Voehringer et al., 2001; Cyktor et al., 2013; Simon et al., 2015
	Induces changes in gene expression, DNA methylation and hypermethylation associated with oxidative stress-induced senescence and increased levels of CXCL9, CXCL10 and TNF-α	Bobak et al., 2022
	Induces epigenetic changes in transcription factors, such as NFKBIA, TCF7, CIITA, MYC, NFAT and DNMT1/3A	Torres et al., 2018
***Helicobacter hepaticus***	CdtB is associated with increased senescence markers, such as SA-β-Gal, p21, and p53	Guerra et al., 2011; Guidi et al., 2013; Péré-Védrenne et al., 2017
***Helicobacter pylori***	Promotes redox imbalances, DNA damage, inflammation and epigenetic changes	Handa et al., 2010; Hanada et al., 2014; Valenzuela et al., 2015
	CagA leads to the up-regulation of p21	Terebiznik et al., 2009; Lewinska and Wnuk, 2017; Aguilera et al., 2018
***Pseudomonas aeruginosa***	Induces senescence in macrophages exhibiting decreased phagocytosis ability	Li et al., 2018
***Porphyromonas gingivalis***	Elevated expression of senescent cellular markers in immune cells	Elsayed et al., 2021
	Increased expression of SA-β-Gal, p16, p53, and p21^Waf1/Clip1^, among others	Elsayed et al., 2021
	Induces cellular senescence through the secretion of bacterial metabolite butyrate	Okumura et al., 2021
***Porphyromonas asaccharolytica***	Induces cellular senescence through the secretion of bacterial metabolite butyrate	Okumura et al., 2021
***Lactobacillus fermentum***	Plays a protective role against senescence in a model of premature hydrogen peroxide-induced senescence by limiting DNA damage, SASP activation, and stress-induced stimulation of PI3K/Akt/mTOR pathway	Kumar et al., 2020

### 4.3 Bacterial infections

**Figure 3:**
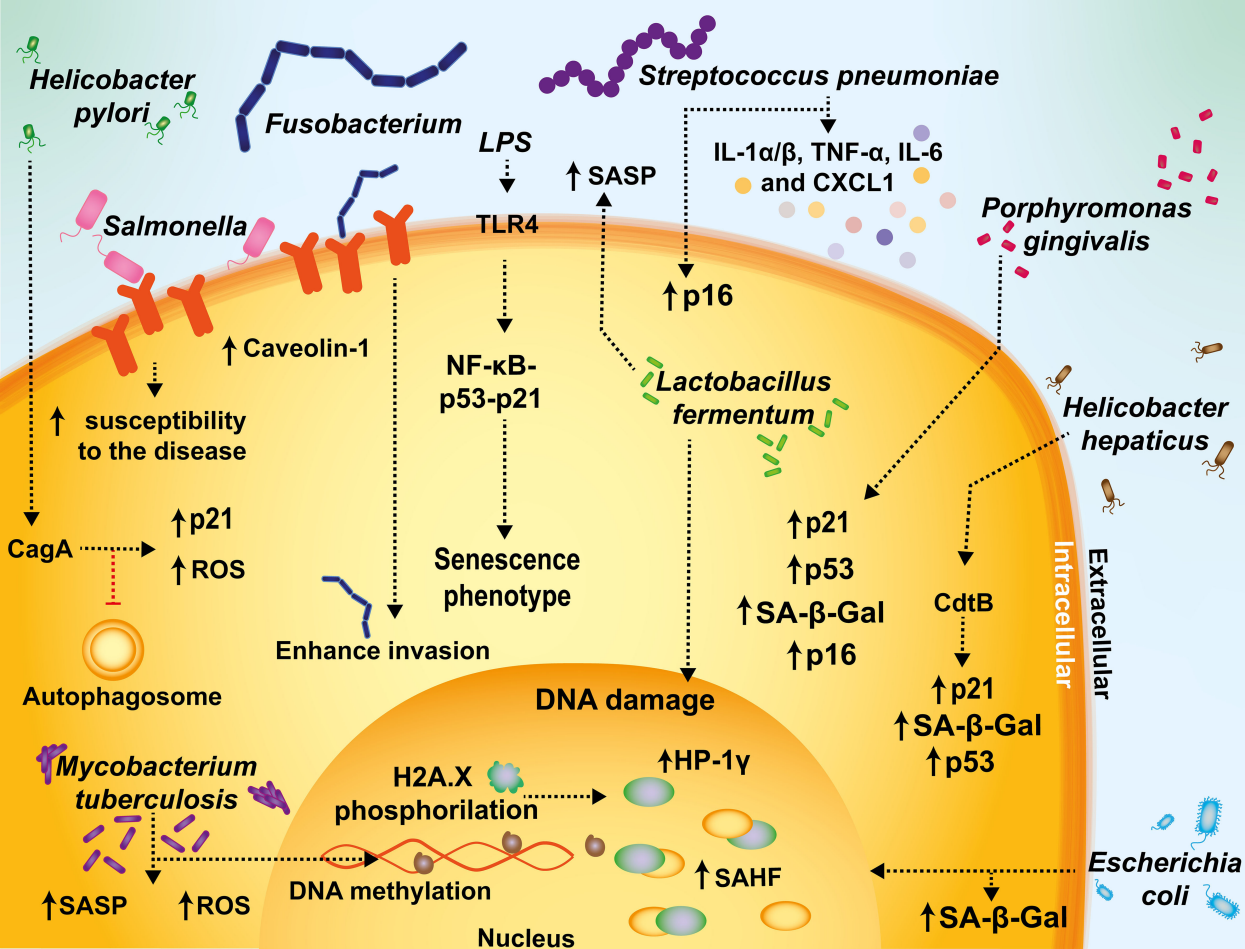
Mechanisms mediating senescence interplay with bacterial infections. *Fusobacterium* and *Salmonella* increase caveolin-1 expression and promote bacterial uptake. During *Streptococcus pneumoniae* infections, inflammation is associated with increased senescence markers, such as p16, IL-1α/β, TNF-α, IL-6, and CXCL1. In sepsis or endotoxemia, the activation of the activation of TLR4 by lipopolysaccharide (LPS) sensing can promote a senescent phenotype in infected cells through the NF-kB-p53-p21 axis, supporting a prolonged inflammatory status with long-term adverse outcomes. *Escherichia coli*-infected cells display cellular markers related to senescence, such as SA-β-Gal activity and senescence-associated heterochromatic foci (SAHF). *Helicobacter pylori* has been described to have the ability to activate senescence and inhibit autophagosome maturation in T cells. *M. tuberculosis* induces changes in gene expression, DNA methylation and hypermethylation, and an increase in ROS and SASP. The CdtB of *Helicobacter* is associated with increased senescence, as evidenced by SA-β-Gal, p21, and p53. *Helicobacter pylori* cytotoxin-associated gen A (CagA) induces p21, that in turn induces cellular senescence. *Porphyromonas gingivalis* is associated with an increased expression of SA-β-Gal, p16, p53, and p21. *Lactobacillus fermentum* has been reported to play a protective role against senescence in a model of premature hydrogen peroxide-induced senescence by limiting DNA damage and SASP activation.

Regarding bacterial infections and senescence, caveolin-1, has been reported to enhance the infection of *Fusobacterium nucleatum* in senescent gingival fibroblasts (Ahn et al., 2017) (**Figure 3**), and in the context of respiratory tract infections *Streptococcus pneumoniae* inflammation was associated with lung from aged mice that were in turn linked with an increase in senescence markers, such as p16, IL-1α/β, TNF-α, IL-6, and CXCL1, that coincided with an augmented expression of a *S. pneumoniae* ligand on the cell surface (Shivshankar et al., 2011) (**Figure 3**).

Reciprocally, bacterial infections have also been associated with the induction of senescence. For instance, bacterial infections have been reported to induce senescence by a persistent inflammatory response, with pathogen-associated molecular patterns (PAMPs), such as bacterial LPS being related to the induction of stem cell senescence via the activation of the pattern recognition receptor TLR4, and through the NFκB-p53-p21 signaling axis (Feng et al., 2018; Fan et al., 2022) (**Figure 3**). Although *Escherichia coli*-infected cells may undergo cell death, some of these cells display cellular markers related to senescence, such as the formation of PML bodies, SA-β-Gal activity and SAHF (Secher et al., 2013) (**Figure 3**). Additionally, bacterial genotoxins, such as those belonging to the family of cytolethal distending toxins (CDTs) that are encoded by Gram-negative pathogenic bacteria, have been reported to have their CDT nuclease activity involved in inducing double-strand and single-strand breaks in host DNA, triggering a DNA damage response and cell-cycle arrest, as well as cellular distension (Cortes-Bratti et al., 2001; Fedor et al., 2013). Consistently, the downregulation of TLR4 reduced this phenotype in infected cells (Yoon et al., 2022, 4). Furthermore, in a recent report (Merdji et al., 2021), it was shown that septic shock induces prolonged endothelial and vascular senescence in Wistar male rats, with a pro-inflammatory status remaining up to 90 days after septic challenge, which increased the long-term risk for adverse cardiovascular events.

Importantly, there is recent evidence that suggests that chronic infections caused by *Mycobacterium tuberculosis* are affected, at least in part, by senescence. *In vivo* analyses with murine models showed that the senescence marker known as killer cell lectin-like receptor G1 (KLRG1), is expressed at higher levels in T cells upon *M. tuberculosis* infection during aging and decreases after pharmacologically treating tuberculosis. This finding suggests a correlation between KLRG1 expression and disease progression (Voehringer et al., 2001; Cyktor et al., 2013; Simon et al., 2015). On the other hand, some studies have proposed that immunosenescence plays an important role in age-associated reactivation of tuberculosis and that KLRG1 is involved in this phenomenon, as evidenced using KLRG1 knock-out mice (Aguilera et al., 2018). Studies conducted in guinea pigs and humans have shown that *M. tuberculosis* induces changes in gene expression, DNA methylation and hypermethylation, which are associated with oxidative stress-induced senescence with increased levels of CXCL9, CXCL10 and TNF-α (Bobak et al., 2022) (**Figure 3**). Interestingly, these studies reported that both, guinea pigs and aging humans infected with *M. tuberculosis* display similar epigenetic changes in transcription factors, such as NFKBIA, TCF7, CIITA, MYC, NFAT and DNMT1/3A (Torres et al., 2018). Lastly, a study carried out in old mice found that having aged memory CD8^+^ T cells translated into reduced *M. tuberculosis* control (Piergallini and Turner, 2019).

Another example of a chronic bacterial infection in which senescence has been observed is that mediated by *Helicobacter hepaticus*. Interestingly, recent reports suggest that the expression of the subunit of the cytolethal distending toxin of this bacterium, termed CdtB, is associated with increased senescence, as evidenced by the senescence markers SA-β-Gal, p21, and p53 (Guerra et al., 2011; Guidi et al., 2013; Péré-Védrenne et al., 2017). On the other hand, *Helicobacter pylori* cytotoxin-associated gen A (CagA) has been reported to lead to the up-regulation of p21 in an ERK-dependent manner, that in turn induces cellular senescence (Terebiznik et al., 2009; Lewinska and Wnuk, 2017; Aguilera et al., 2018) (**Figure 3**).

*H. pylori* is capable of surviving outside the gastrointestinal tract and has been related to some skin chronic infections associated with senescence promoting redox imbalances (Handa et al., 2010), DNA damage (Hanada et al., 2014), inflammation and epigenetic changes in host cells that may provoke extragastric manifestations (Valenzuela et al., 2015), all of which are triggers and biomarkers of cellular senescence (Lewinska and Wnuk, 2017).

*Pseudomonas aeruginosa* has been reported to induce senescence in macrophages exhibiting decreased phagocytosis ability. This senescence process was reported to be regulated by interactions between NADPH oxidase gp91phox and NF-κB p65 via ROS as a second messenger (Li et al., 2018). *Porphyromonas gingivalis* is another pathogen associated with elevated expression of senescent cellular markers in immune cells, such as DCs with bacterial invasion leading to an increase in the secretion of inflammatory exosomes which in turn amplify immune senescence in paracrine to bystander cells (Elsayed et al., 2021). Additionally, there is an increased expression of SA-β-Gal, p16, p53, and p21^Waf1/Clip1^, among others (Elsayed et al., 2021) (**Figure 3**). Importantly, *Porphyromonas gingivalis* and *Porphyromonas asaccharolytica* can induce cellular senescence through the secretion of the bacterial metabolite butyrate, and an unbalanced increase of these butyrate-producing bacteria has been associated with colorectal tumorigenesis (Okumura et al., 2021).

Finally, the probiotic bacterium *Lactobacillus fermentum* has been reported to play a protective role against senescence in a model of premature hydrogen peroxide-induced senescence of murine preadipocyte cell line (3T3-L1), by limiting DNA damage, SASP activation, and stress-induced stimulation of PI3K/Akt/mTOR pathway in these cells, which highlights the potential of using probiotics as pro-longevity strategies (Kumar et al., 2020) (**Figure 3**).

An increasing amount of evidence indicates that pathogenic bacterial infections induce senescence in host cells, and that some harmless bacteria can actually counteract these effects. It will be interesting to know whether treatment with antibiotics can limit bacteria-induced senescence and the contribution of bacterial components, i.e. dead bacteria, to senescence in related studies, as this has not been assessed extensively. Furthermore, it will be important to acquire increased knowledge on the effects of commensal bacteria over senescence and potential counteractions as reported for probiotic bacteria in order to determine the molecular determinants that govern this phenomenon, mainly in host tissues at the interphase with the environment.

Information regarding bacterial infections and their interplay with senescence is summarized in **Table 1**.

## 5 Inmunosenescence

To better understand the relationship between cellular senescence and infections it is also important to assess cell aging in the context of immune cells that directly and indirectly interact with pathogens. Senescence-related changes affect various components of the innate and adaptive immune system and overall may result in a state of immunodeficiency (Akbar and Fletcher, 2005). Below, we revise aspects related to the impact of senescence over different components of the immune system, including innate immune components, T cells and B cells for a better understanding of its effects over infections.

### 5.1 Impact of immunosenescence on innate immune cells

**Figure 4:**
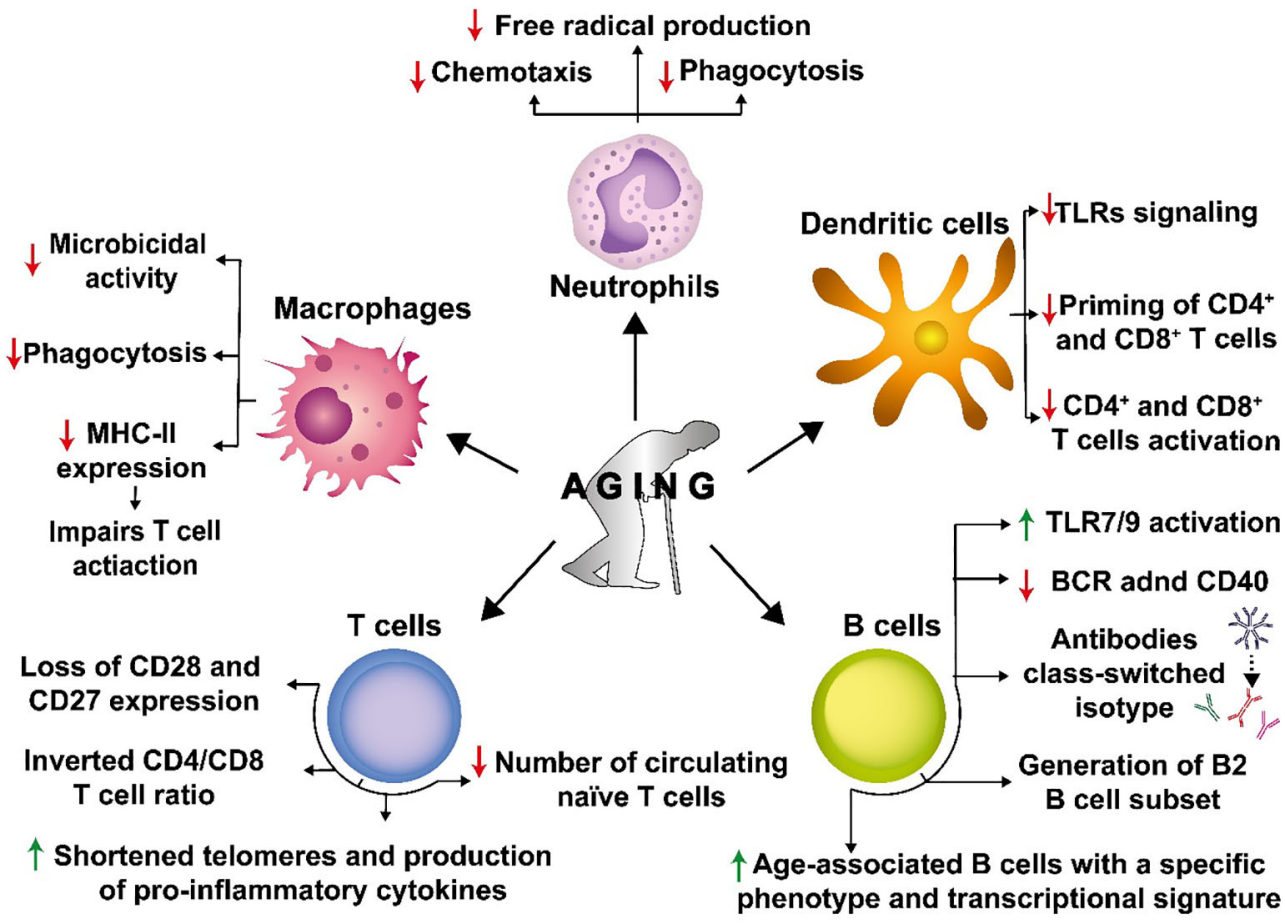
Senescence causes a detrimental impact on the immune system. Senescence affects numerous innate and adaptive immune cells. Senescence decreases chemotaxis, phagocytosis, and free radical production in neutrophils, which are necessary to engulf and kill different pathogens. Macrophages also experience decreased phagocytosis and reduced expression of major histocompatibility complex II molecules (MHC-II), affecting their microbicidal capacities and contributing to impaired T cell activation. Dendritic cells (DCs) display a reduced expression of TLRs, leading to reduced CD4^+^ and CD8^+^ T cell priming and activation. In T cells, senescence induces the loss of CD28 and CD27 expression on the cell surface and alters the CD4/CD8 T cell ratio. Additionally, replicative senescence in T cells is associated with shortened telomeres, increased production of pro-inflammatory cytokines, and a reduced number of circulating naive cells. In B cells, senescence induces an antibody class-switch that results in decreased immunity and the generation of B cells of the B2 cell subset that are short-lived recirculating cells that are metabolically impaired. In addition, there is an increase in the age-associated B cell subset with a specific phenotypic and transcriptional signature in the splenic B cell compartment.

Innate immune cells, such as neutrophils, macrophages, dendritic cells (DCs) and, natural killer (NK) cells, are the first line of defense against pathogens, which may be responsible for limiting the spread of infection and, more importantly, play a key role in promoting the initiation of an adaptive immune response against these agents (Stegelmeier et al., 2019). Several reports indicate that innate immune cell functions are altered with aging (Solana et al., 2012). Indeed, different studies have reported that aging affects neutrophil function by impairing chemotaxis, phagocytosis, and free radical production, all of which are necessary for engulfing and eliminating some pathogens such as bacteria (Butcher et al., 2001; Hazeldine and Lord, 2015) (**Figure 4**). Macrophages also experience reduced phagocytic capacity and decreased expression of MHC class II with aging (Plowden et al., 2004), which affects their microbicidal activity and impairs T cell activation (Villanueva et al., 1990; Plowden et al., 2004) (**Figure 4**).

Likewise, aging impacts DCs, which are key professional antigen-presenting cells (APCs) responsible for bridging innate and adaptive immune responses (Roney, 2019). Upon aging, DCs display a reduced expression of Toll-like receptors (TLRs), which limits their maturation and likely their potential for priming naïve T cells (Jing et al., 2009) (**Figure 4**). Indeed, it has been shown that aging reduces DC-mediated priming of CD4^+^ and CD8^+^ T cells (Pereira et al., 2011) (Sridharan et al., 2011) (**Figure 4**). The reduction of CD4^+^ T cell activation will harm T cell-dependent humoral responses, which in addition to the adverse effects of aging over B cells, will significantly reduce the host’s antibody responses.

### 5.2 Impact of immunosenescence over T cell immunity in the context of infections

T cells and their subpopulations are key cellular components of the adaptive immune system and are derived from hematopoietic stem cells from the bone marrow (Janeway, 2001). The almost exclusive antigen-specificity of each T cell allows the organism to respond to a virtual infinite number of foreign antigens and/or pathogens, which may be encountered throughout the individual’s life (Janeway, 2001; Moser and Leo, 2010). With this broad capacity to recognize numerous antigens, the number of cells that can recognize and respond to any individual antigen is highly limited (Rosenblum et al., 2016). Therefore, to produce sufficient specific effector cells to fight an infection, an activated T cell must proliferate so that its progeny exerts effector cells in enough numbers to affect the pathogen effectively (Hilleman, 2004). For this reason, a limitation in the number of cell divisions, or associated processes, could potentially impair immune function against pathogens (Effros, 1998). This deterioration of the immune system can lead to increased susceptibility to infections, reduced effectiveness of vaccines, and possibly an increased occurrence of several diseases in the elderly (Renshaw et al., 2002; Pera et al., 2015).

An important biomedical development in the last century is the generation of vaccines, which have prevented the spread of infectious diseases, eradicated some of them, and saved millions of lives (Tomori, 2011). Vaccines have been the main asset to controlling and preventing COVID-19 (Remmel, 2021). When analyzing the effectivity of vaccines in terms of age-ranges in the population, their efficacy decreases with age, with increased infection susceptibility in the elderly, which occurs similarly for other viral and bacterial infections upon vaccination (Nicoletti et al., 1993; Crooke et al., 2019). These alterations may be related to immunosenescence.

Although studies regarding vaccines against pathogens such as influenza have shown mixed results in effectivity throughout age groups, most of these studies reveal that the antibody responses of young individuals are more robust as compared to those of older individuals (Mosterín Höpping et al., 2016). At the cellular level, this has been attributed to the DC-T cell axis, as reported by Panda et al. (2010). Furthermore, defective TLR signaling in primary human DCs from elderly individuals led to a decreased production of proinflammatory cytokines, which was associated with a weaker antibody response to influenza vaccination (Lefebvre et al., 2016) (**Figure 4**). Moreover, pathogens have several virulence factors that may induce senescence hallmarks directly or indirectly in host cells (Redeker et al., 2017). Furthermore, the extended lifespan of humans has been associated with detriments to the immune system, and it has been recently demonstrated that pathogens may use senescent cells to establish persistent infections in older organisms (Fülöp et al., 2013).

Some of these alterations can be attributed to a phenomenon known as T cell replicative senescence (Chou and Effros, 2013), a stage characterized by dysregulated immune function with the loss of the expression of costimulatory molecules in T cells, namely CD28 and CD27 (Weiskopf et al., 2009) (**Figure 4**). These are important molecules for promoting cell proliferation and the survival of CD8^+^ T cells (Effros et al., 2005, 8). T cell replicative senescence is also associated with shortened telomeres and increased production of proinflammatory cytokines (Effros, 2004) (**Figure 4**). These alterations are related to phenotypic changes in CD8^+^ T lymphocytes. It has been shown that senescent CD8^+^ T cells, which tend to accumulate in the elderly, often have antigenic specificity against cytomegalovirus (CMV), suggesting that this common and persistent infection may contribute to driving immune senescence and causing functional and phenotypic changes in T cells compartment. Impaired T cell function has been pointed out as an important factor in responses to pathogens upon aging.

Consequently, a host may display with aging a reduced capacity to respond to recurrent infections (Dock and Effros, 2011; Fülöp et al., 2013, 8). T cells in old age have also been identified in patients with chronic infections, such as with the HIV (Miller, 1996; Akbar and Fletcher, 2005). Clinically, these defects correlate with increased morbidity and mortality in the elderly upon infectious diseases (Vicente et al., 2016). Furthermore, upon aging changes in T cell differentiation, when compared to younger individuals, shows a bias towards the formation of a T follicular helper phenotype, instead of a Th1 phenotype (Lefebvre et al., 2016). A Th1 phenotype is generally essential for viral clearance, so a decrease in these cells will directly impact infection resolution (Lefebvre et al., 2016). Altogether, these T cell alterations will affect B cell activation, their differentiation and antibody production. Indeed, analyses of plasma cells revealed that antibody production was affected at the levels of antigen-specific antibodies to be produced upon vaccination, and not as a reduction in antibody avidity, which correlated with the phenotype bias of T helper cells (Sasaki et al., 2011).

The main question regarding the occurrence of the above-described immunological effects seems to be related to the processing and presentation of antigens (Pamer, 1999), as well as to environmental cytokines, as these can affect both, ongoing immune responses and the maintenance of lymphoid populations (Ku et al., 2000; Akbar et al., 2016).

Immunosenescence includes another primary feature that relates to impaired hematopoietic stem cell function, such as decreased numbers of circulating naïve T cells from aged mice as compared to young mice, and an inverted CD4/CD8 ratio, together with increased levels of proinflammatory cytokines, such as IL- 6 and TNF-α (**Figure 4**) (Deeks, 2011; Jiang et al., 2013). There is also evidence regarding an alteration in the profile of CD4^+^ T cells (Th), from a Th1 phenotype toward Th2, which may result in a shift in the balance between humoral and cell-mediated immune responses to pathogens (Linton, 2001; Spellberg and Edwards, 2001). Therefore, pathogens such as influenza, pneumococcus, and varicella-zoster virus (VZV), and emerging pathogens, such as West Nile virus (WNV) and severe acute respiratory syndrome (SARS)-associated coronavirus, may have a disproportionate symptomatology with high incidence and deaths in the elderly, as immunosenescence is more commonly seen in the affected when compared with healthy younger individuals (Yancik and Ries, 2000; Lin and Bhattacharyya, 2012).

Additionally, it is also possible that an aging immune system is inefficient in controlling latent virus reactivation (and gene expression), due to the intrinsic effects of senescence over T cells, which would allow prolonged persistence of viral antigens and continuous stimulation of virus-specific T cells leading to their exhaustion (Smithey et al., 2012; Fülöp et al., 2013).

### 5.3 B cells immunosenescence

B cells are an essential component of the adaptive immune system and are specialized in producing antibodies (Burton et al., 2000). For most antigens requiring high-affinity antibodies, the activation of B cells requires the help of CD4^+^ T cells. In this process, B cells uptake antigens through endocytosis by through their B cell receptors (BCRs, which later will be secreted as soluble antibodies), to further process and present their derived antigens in MHC-II molecules when peptidic in nature (Crotty, 2011). The peptide-MHC-II molecules interact with the T cell receptor (TCR) on the surface of antigen-specific Th cells (Crotty, 2011). If activating, this peptide-MHC-II-TCR engagement will promote an increase in the expression of co-activating molecules, such as CD40, CD40-L, and ICOS, and cytokines such as IL-21, IL-4, BAFF, and IFN-γ (93). These signals may promote B cell activation, inducing their proliferation, the initiation of the germinal center reaction, and antibody isotype switch (Vinuesa et al., 2005). The antibody isotype switch is the process whereby activated B cell change the type of antibody produced, from IgM to either IgG, IgE, or IgA depending on the functional requirements of such antibodies (Roco et al., 2019) (**Figure 4**). These changes are critical for an effective humoral immune response, and several are affected during aging. Antibody class-switch is a process sustained by the enzyme activation-induced cytidine deaminase (AID) (Stavnezer, 2011). Some reports indicate no difference in the expression of AID B cells from young and old mice (Russell Knode et al., 2019). However, mitogen-stimulated B cells from aged mice generate low levels of class-switched antibodies (Frasca et al., 2004), which was attributed to a reduced expression of AID and E47, the latter belonging to a set of proteins that regulate AID expression (Frasca et al., 2004).

The impact of aging on humoral immunity is linked to several biological processes in B cells, which have been reported in humans and mice (Frasca et al., 2005; Buffa et al., 2011). For instance, maturation and differentiation of B cells from precursors in the bone marrow are necessary to maintain the proportion of different subsets of B cells in various tissues, in a process that is in contrast to what happens to T cells (Alter-Wolf et al., 2009). In this regard, it has been reported that the B2 B cell subset is poorly generated in aged mice (Alter-Wolf et al., 2009) (**Figure 4**). This B cell subset is short-lived, metabolically quiescent, involves follicular and marginal B cells, and is the primary subtype of B cells that responds to protein antigens (Alter-Wolf et al., 2009). One of the main signals involved in this process is the availability of IL-7 produced by stromal cells, and since the bone marrow microenvironment is altered in aged mice, this leads to a reduced production of this cytokine by stromal cells (Stephan et al., 1998). In addition, age-associated B cells have been identified as a subset of B cells with a specific phenotype and transcriptional signature, which continuously increase in the splenic B cell compartment of old mice and depends on TLR7 and TLR9 activation, but not on BCR engagement and the costimulatory molecule CD40 (Hao et al., 2011) (**Figure 4**). These cells have also been detected in young lupus-prone NZB/WF1 mice and peripheral blood samples of elderly women with autoimmune diseases (Cancro, 2020). Antibody production also changes during aging, with several reports indicating that older individuals’ antibodies are less protective than younger individuals (Nicoletti et al., 1993; Howard et al., 2006).

The importance of highlighting the detrimental effect of aging on antibody production relies on the susceptibility of older individuals to infection by different pathogens, which leads to a significant severity of disease and increased mortality, a matter of great concern, especially considering that globally, people are living longer (Keilman, 2021)

## 6 Future perspectives

Considering the studies reported to date in the field of senescence and infections and the accelerated global growth of the population 60 years and older, it will be paramount to focus current findings related to cellular senescence and aging towards the search of new therapeutic perspectives, or strategies for the prevention, or reversal of the diverse array of pathologies associated with aging. Importantly, numerous studies show that the selective elimination of senescent cells promotes a better prognosis of diseases, and thus, senomorphic strategies for treating these diseases are currently under development and should enter in the short-term clinical studies to ultimately reach those in need. However, even though it is known that viral and bacterial infections promote cellular senescence, there are no guidelines related to cumulative senescence in tissues, or formal strategies oriented at modulating the senescent phenotypes of cells induced by pathogen infection. Additionally, it will be important that a fraction of the efforts in the field of aging, immunology and pathophysiology be focused on the overall damaging or beneficial effects for the host of senescence and immunosenescence. Another important question that remains to be addressed is the duration of the senescence and immunosenescence phenotypes elicited by pathogen infections over host cells, and to determine whether these are long-lasting, or eventually acute. Noteworthy, the effects of vaccines and treatments against infection-induced cellular senescence should be studied in more detail to assess the contribution of these approaches to this phenomenon and potential benefits.

## 7 Concluding remarks

The role of senescence in an organism is somewhat dual, as this cellular process has been described to have both, beneficial and detrimental effects on the individual. It can act as a potent anticancer mechanism and attenuate some diseases, such as liver fibrosis, renal fibrosis, and atherosclerosis. However, an accumulation of senescent cells has been associated with pulmonary fibrosis and type-2 diabetes, among others. It can also harm the immune system, downregulating its response capacity upon infections and contribute to autoimmune diseases. Thus, the regulation of senescence may have a therapeutic potential by balancing senescent and non-senescent processes needed to maintain cellular homeostasis and health. This remains to be determined in the context of pathogen infections.

Immunosenescence has been reported to participate in several processes of the immune system and affect a wide range of antimicrobial functions within the host. Additionally, the effectivity of vaccines is also affected by aging, as a decrease in their prophylactic effects have been seen in older individuals being vaccinated as compared to younger people. This condition, in turn, can lead to a higher susceptibility of the host to infections caused by different pathogens and more severe diseases, which is a significant concern for public health systems worldwide due to increasing life expectancy. For instance, in the recent COVID-19 pandemic, older individuals were more susceptible to develop severe symptoms than younger individuals. Interestingly, the role of senescence in infections is a fast growing field with multiple studies emerging in this area since last year (Marrella et al., 2022).

Furthermore, some pathogens modulate senescence for their own benefit. Some pathogens have evolved molecular mechanisms to induce senescence and use this cellular process on their favor for carrying out an effective replication cycle. However, other pathogens have evolved mechanisms to induce senescence and use this cellular process for their benefit. Additionally, a weakened immunosenescent immune system may also allow latent viruses to reactivate easier or more frequently, causing new rounds of infection in the host and virus shedding. Thus, regulating this cellular state may be a potential future therapeutic target for treating some infectious diseases. However, the scope of this Review does not consider how senolytic or senomorphic drugs may be used as a therapeutic target against infections, as this has been described to some extent in other articles (Lee et al., 2021; Marrella et al., 2022). Nevertheless, this should be managed with precaution, as an imbalance between senescent and not senescent cells in the organism may be detrimental to relevant cellular and tissue processes and functions that are needed for homeostasis.

## Author contributions

All authors contributed equally on the writing and revision of this article. AR, CA, LR-G, CF, MC and PG contributed on the preparation and revision of the figures. All authors contributed to the article and approved the submitted version.
